# PRMT5 Associates With the FOXP3 Homomer and When Disabled Enhances Targeted p185^erbB2/neu^ Tumor Immunotherapy

**DOI:** 10.3389/fimmu.2019.00174

**Published:** 2019-02-08

**Authors:** Yasuhiro Nagai, Mei Q. Ji, Fuxiang Zhu, Yan Xiao, Yukinori Tanaka, Taku Kambayashi, Shigeyoshi Fujimoto, Michael M. Goldberg, Hongtao Zhang, Bin Li, Takuya Ohtani, Mark I. Greene

**Affiliations:** ^1^Department of Pathology and Laboratory Medicine, Perelman School of Medicine, University of Pennsylvania, Philadelphia, PA, United States; ^2^Unit of Molecular Immunology, Key Laboratory of Molecular Virology & Immunology, CAS Center for Excellence in Molecular Cell Science, Institute Pasteur of Shanghai, Shanghai Institutes for Biological Sciences, Chinese Academy of Sciences, Shanghai, China; ^3^Adviser of Seishin Medical Group, Takara Clinic, Tokyo, Japan; ^4^Macrophage Therapeutics, Englewood Cliffs, NJ, United States; ^5^The Department of Immunology and Microbiology & Shanghai, Institute of Immunology, Shanghai JiaoTong University School of Medicine, Shanghai, China; ^6^Penn Institute for Immunology, Perelman School of Medicine, University of Pennsylvania, Philadelphia, PA, United States

**Keywords:** PRMT5, FOXP3 regulatory T cells, autoimmunity, scurfy, tumor immunity, breast cancer

## Abstract

Regulatory T cells (Tregs) are a subpopulation of T cells that are specialized in suppressing immune responses. Here we show that the arginine methyl transferase protein PRMT5 can complex with FOXP3 transcription factors in Tregs. Mice with conditional knock out (cKO) of PRMT5 expression in Tregs develop severe scurfy-like autoimmunity. In these PRMT5 cKO mice, the spleen has reduced numbers of Tregs, but normal numbers of Tregs are found in the peripheral lymph nodes. These peripheral Tregs that lack PRMT5, however, display a limited suppressive function. Mass spectrometric analysis showed that FOXP3 can be di-methylated at positions R27, R51, and R146. A point mutation of Arginine (R) 51 to Lysine (K) led to defective suppressive functions in human CD4 T cells. Pharmacological inhibition of PRMT5 by DS-437 also reduced human Treg functions and inhibited the methylation of FOXP3. In addition, DS-437 significantly enhanced the anti-tumor effects of anti-erbB2/neu monoclonal antibody targeted therapy in Balb/c mice bearing CT26Her2 tumors by inhibiting Treg function and induction of tumor immunity. Controlling PRMT5 activity is a promising strategy for cancer therapy in situations where host immunity against tumors is attenuated in a FOXP3 dependent manner.

## Introduction

Regulatory T cells (Tregs) limit autoimmune processes directed at self-antigens. While the Treg's immunosuppressive functions are beneficial to limit autoimmune processes, studies examining certain syngeneic tumors in animals have confirmed a detrimental role for Tregs in cancer (1). In human tumors, infiltration of tumor sites with Foxp3 Treg cells can be associated with a less favorable prognosis (2, 3). Thus, targeting Tregs in tumor bearing hosts represents a strategy for tumor immunotherapy (4–6) although studies in some human tumor tissues have suggested FOXP3 effects only as a contributory, but not a deterministic role, on tumor malignant growth (7).

FOXP3 is a dimeric and/or tetrameric transcription factor in Tregs and fulfills an important role in both Treg development and function (8, 9). FOXP3 forms an ensemble with several proteins, many of which have been shown to play roles in enhancing or modifying FOXP3 activities. These include several epigenetic modulators for FOXP3, such as the histone acetyltransferases Tip60 (KAT5) and p300 (KAT3b) that acetylate FOXP3 at discrete lysines and enhance Treg function (10–12) through stabilization and limiting degradation caused by FOXP3 ubiquitination (13). In contrast, histone deacetylases (HDAC7) can remove the acetyl groups, and this process promotes degradation of FOXP3. Phosphorylation of FOXP3 at serine 418 is also important for Treg function (14). Phosphorylation of FOXP3 by the Pim1 and Pim2 serine/threonine kinases diminishes FOXP3 functions (15, 16). Thus, post-translational modifications (PTM) of FOXP3 are important for modifying Treg functions and stabilities. In an effort to characterize other PTM proteins of FOXP3, we have identified Protein Arginine Methyl Transferase 5 (PRMT5) as a novel PTM protein that modifies FOXP3 functions by arginine methylation.

## Materials and Methods

### Plasmid and Antibodies

All human PRMT cDNAs were purchased from OpenBiosystem and GE Dharmacon, and subcloned into the mammalian expression vectors *pIRESpuromycin-HA2* with two HA eptitope tags or *pIRESpuromycin-FLAG2* with two FLAG eptitope tags, and HA2-or FLAG2-Foxp3 vector was generated as previously described (10). PRMT5 shRNA vector was obtained from TRC shRNA vector library (GE Dharmacon). The sequence is below: TATTCCAGGGAGTTCTTGAGG (shPRMT5 85); ATAAGGCATCTCAAACTGGGC (shPRMT5 86). For the point mutation of Foxp3, Quick change II site-directed mutagenesis kit (Agilent) was used per manufacturer's instructions.

### Mice

To generate the PRMT5^fl/fl^ mouse, PRMT5 conditionally targeted ES cells were obtained from the International Mouse Phenotyping Consortium (Prmt5^tm2a(EUCOMM)Wtsi^). In the targeted cells, Exon 6, which encodes the catalytic domain, is sandwiched by two loxp sites, and lacZ reporter and Neomycin genes are inserted upstream together with two FRT sequences. We injected the ES cells into C57BL/6 blastocysts and obtained chimeric animals. The founder animals were mated with flippase transgenic mice (B6.Cg-Tg (ACTFLPe)^9250Dym/J^, 005703, Jackson Lab) to delete lacZ and Neomycin genes. Foxp3^Creyfp^ (B6.129(Cg)-Foxp3^tm4(YFP/Cre)Ayr^/J, 016959) and CD4^cre^ (Tg(Cd4-cre)1Cwi/BfluJ, 017336) mice were obtained from Jackson Laboratory. All animals were housed and bred in a specific pathogen-free animal facility of the University of Pennsylvania. All the experiments were performed following national, state, and institutional guidelines. Animal protocols were approved by the University of Pennsylvania Institutional Animal Care and Use Committee.

### Cell Culture and Transfection

293T cells were grown in DMEM supplemented with 10% heat inactivated fetal bovine serum and antibiotics (1% penicillin/streptomycin; Invitrogen) at 37°C in a humidified incubator with 5% CO2 (v/v). Cells were grown to 80% confluency in 6-well plates, and transient transfection was carried out using a mixture of 6 μg DNA and 18 μl FuGENE 6 (Roche) according to manufacturer's instructions. Twenty-four hours after transfection, the cells were lysed with high salt lysis buffer [20 mM Tris-Cl pH 7.5, 420 mM NaCl, 1% TritonX-100, and complete mini protease inhibitor cocktail (Roche)], then prepared for western blot analysis. For the PRMT5 inhibitor treatments cells were transfected with HA-Foxp3 vector and cultured for 24 h. Then inhibitors were added to the cells with indicated concentrations of CMP5 (IC50: unavailable, Millipore), DS-437 (IC50: 5.9 μM, Sigma), HLCL-61 (IC50: 7.21-21.46 μM for acute myeloid leukemia cell line), EPZ004777 [IC50: 50 μM for PRMT5 (17)], and EPZ015666 (IC50: 20 nM, Selleckchem) and incubated for 16 h. For T cell culture, RPMI-1640 medium supplemented with 10% FBS, 1X non-essential amino acids (Invitrogen), 2 mM sodium pyruvate (Invitrogen) and 50 μM β-mercaptoethanol (Sigma) was used.

### Mass Spectrometry

293T cells were transfected with FLAG-Foxp3 or empty vectors, lysed with high salt lysis buffer, and then immunoprecipitated with anti-FLAG agarose beads (Sigma) overnight at 4°C. The precipitates were then washed three times with lysis buffer and boiled for 5 min in SDS loading buffer. Samples were analyzed by SDS-PAGE and specific bands were cut and subjected to mass spectrometry by the University of Pennsylvania Proteomics and System Biology Core. For the methylation analysis, 293T cells were transfected with HA-Foxp3 vector and immune precipitated with anti-HA magnetic beads (Thermo FIsher). Proteins were eluted with elution buffer (Thermo Fisher) and concentrated by vivaspin 500 (GE Healthcare). Samples were analyzed by SDS-PAGE and subjected for mass spectrumtry by the CHOP Proteome Core at the University of Pennsylvania.

### Immunoprecipitation and Western Blotting

Cells were lysed in lysis buffer and the soluble fractions were collected and incubated with anti-HA angarose, anti-FLAG agarose (Sigma-Aldrich), or anti-symmetric dimethyl arginine antibody Sym10 (Upstate) conjugated with Dynabeads protein G magnetic beads (Invitrogen) for 2 h at 4°C. The precipitates were then washed three times with lysis buffer and boiled for 5 min in SDS loading buffer. Samples were analyzed by SDS-PAGE, transferred to Immobilon-P (Millipore) PVDF membrane, and probed with anti-Flag M2-Peroxidase (Sigma), or anti-HA Peroxidase (3F10; Roche). For the detection of tag proteins, immunocomplexes were detected using Immobilon Western Chemiluminescent horseradish peroxidase (HRP) Substrate (Millipore). For human Tregs, expanded cells were harvested and lysed on ice for 1 h with RIPA buffer (50 mM Tris-HCl (pH7.5), 150 mM NaCl, 1% NonidetP-40, 0.25% NaDOC, 1 mM MgCl_2_, and 10% (vol/vol) glycerol) containing protease inhibitor (1:100; P8340; Sigma-Aldrich), NaF (10 mM), and PMSF (1 mM). Cell lysates were cleared by centrifugation, and the supernatants were incubated with anti-FOXP3 (1 μg, eBio7979), anti-PRMT5 (1 μg, Millipore O14744), or IgG (1 μg, 5415S; Cell Signaling) at 4°C overnight, and then immunoprecipated with Protein G-Sepharose beads (P3296; Sigma) for 1 h at 4°C. The immunocomplexes then were washed with RIPA buffer and examined by Western blotting.

### Flow Cytometry

Spleen, axillary and inguinal lymph nodes, and thymus of 18–21 day-old male mice were collected, and single-cell suspensions were made. The cells were stained with L/D aqua (Thermo Fisher Scientific) per manufacturer's instruction for eliminating dead cells. Then their membranes were stained with anti-CD4-percp (100432, Biolegend), CD8-APC/R700 (564983, BD), TCRβ-BV605 (109241, Biolegend), CD25-PE (553866, BD Bioscience), GITR-PE (120208, Biolegend), CD134-PE/Cy7 (119415, Biolegend), CD304-PE-Cy7 (145211, Biolegend), and/or ICOS-PE/Cy7 (313519, Biolegend), CD44-APC-Fire750 (103062, Biolegend), and/or CD62L-PE-Cy7 (104418, Biolegend). After 20 min of incubation on ice, the cells were then fixed with a Foxp3 staining buffer set (eBioscience) and intracellularly stained with CTLA4-BV421 (106312, Biolegend), KI67-BV421 (652411, Biolegend), Foxp3-APC (17-5773-82, eBioscience) and/or Helios-PE (137206, Biolegend), and subjected to flow cytometry using the fluorescence-activated cell sorter (FACS) LSR (BD Biosciences). FACS data were analyzed with FlowJo software (Tree Star). For separating YFP^+/−^ cells, the cells were fixed with 1% paraformaldehyde in PBS for 5 min on ice after membrane staining, then re-fixed using the Foxp3 staining kit, followed by intracellular staining as described above.

For the cytokine expression studies, the spleen cells were harvested and stimulated with PMA (50 ng/ml) and ionomycin (10 μM) and anti-CD3 (0.5 μg/ml) for 6 h with Protein Transport Inhibitor Cocktail (× 500, eBioscience). The cells were then membrane stained with L/D aqua followed by anti-CD4-percpCy5.5 (100434, Biolegend) CD8-AF488 (100723, Biolegend) and fixed with 1% paraformaldehyde in PBS for 10 min on ice and then re-fixed using the Foxp3 staining kit, followed by intracellular staining as described above. After fixation, the cell were stained with anti-IL-4-BV711 (504133, Biolegend), IL-17a-BV421 (506926, Biolegend), IFNγ-PE-cy7 (505826, Biolegend), and Foxp3-APC as described above and analyzed with FACS LSR.

For TILs, tumors were dissected and minced in digestion buffer (0.025 mg/ml liberase TM, 0.05 mg/ml DNAse I (Roche) in RPMI) and then incubated at 37°C with rotating for 30 min. Then cells were filtered through a cell strainer (Falcon). Cells were then stained with anti-CD4-BV785 (100453, Biolegend), CD8-APC/R700 (564983, BD), PD-1-BV421 (135221 BIolegend), TCRβ-BV605, PD-L1-PE (124308, Biolegend), Ly6G-Percp (127654, Biolegend), CD206-PE/Cy7 (141720, Biolegend), CD45-AF488 (103122, Biolegend), F4/80-BUV395 (565614, BD), NKp46-BUV737 (565085, BD), and Foxp3-APC as described above and analyzed with FACS LSR.

### RNA Sequencing

TCRβ^+^ CD4^+^ CD45RB^low^ CD25^high^ cells from CD4^Cre/+^ or CD4^+/+^ PRMT5^fl/fl^ mice were sorted using the FACS Aria II. A total of 100,000 cells per sample were used for mRNA extraction using Trizol (Invitrogen) and RNeasy micro kit (Qiagen) according to manufacturer's instructions. The extracted mRNA was subjected for RNA sequencing and statistics were analyzed by the High Throughput Sequencing Core at CHOP at the University of Pennsylvania. Differentially expressed gene (DEG) was defined as fold change ≥2 and post-probability of equally expressed (PPEE) < 0.05 calculated by EBSeq method.

### Human Treg Expansion

CD4^+^ T cells were provided by the Human Immunology Core at the University of Pennsylvania from healthy donors. CD4^+^ CD25^high^ CD127^low^ Tregs were sorted using the FACS Aria II (BD Bioscience) to a purity >98%. For *in vitro* expansion, the cells were cultured in T cell medium supplemented with recombinant human IL-2 (200 IU/ml, Peprotech) and anti-CD3/CD28 beads at a 1:1 ratio. The media was changed every other day.

### Lentivirus Production and Transfection to Human Tregs

Lentivirus was produced using the ViraSafe^TM^ lentivirus packaging system (Cell Biolabs) per manufacturer's instructions. Viral supernatants were concentrated by adding 50% PEG8000 and 1.5 M NaCl (Final concentration: 5% PEG8000 and 0.15 M NaCl), rotated overnight at 4°C, then centrifuged for 30 min at 3,300 g. The pellets were suspended in T cell media and used for lentivirus transfection. For lentivirus transfection to human Tregs, expanded human Tregs (3 × 10^5^ cells) were harvested and suspended in lentivirus-containing medium with IL-2 (200 IU/ml) and anti-CD3/CD28 beads in 5 ml round bottom tubes (Fisher) and then cenreifuged for 2.5 h at 450 g at RT. The cells were then re-suspended in T cell medium supplemented with IL-2 (200 IU/ml) and cultured in a 12 well-plate. After 2 days of culture, medium was changed and puromycin (1 μg/ml) was added for selecting transfected cells. After 6 days, live cells were sorted and used for the *in vitro* suppression assay as described below.

### Retrovirus Production and Transfection to Human CD4^+^ Tcells

Foxp3-introduced MIGR1 vectors were used for retrovirus production with pMD2. G and pUMVC (Addgene) at 7.5:5:2.5 ratio using 293T cells. Viral supernatants were concentrated as described above. CD4^+^ T cells were transfected with the same method as lentivirus transfection as described above. Two days after transfection, GFP^+^ cells were sorted and expanded for 5 days. Then GFP^+^ cells were re-sorted and subjected to the suppression assay as described below.

### Treg Suppression Assays

For the mouse cells, CD4^+^ T cells were enriched from splenocytes using a mouse CD4^+^ T cell isolation kit (Stem Cells). CD4^+^ CD25^−^ CD45RB^high^ Teff cells and CD4^+^ CD25^+^ YFP^+^ CD45RB^low^ Treg cells were separated from CD4^+^ cells using the FACSAria II (BD Biosciences). Teff cells were labeled with Cell Trace Violet (CTV, Molecular Probes) and mixed with Tregs and Dynabeads® Mouse T-Activator CD3/CD28 (Life Technologies) in a V-bottom 96-well plate (20,000 cells/well of Teffs, indicated ratio of Tregs, and 0.2 μl/well of CD3/CD28 beads). For the human cells, healthy donor PBMC were provided by the Human Immunology Core of the University of Pennsylvania, and CD4^+^ T cells were enriched using the MACS CD4^+^ isolation kit (Miltenyi Biotech). Then CD4^+^ CD25^−^ CD127^high^ Teff cells and CD4^+^ CD25^+^ CD127^low^ Treg cells were separated from CD4^+^ cells using the FACSAria II (BD Biosciences). Teff cells were labeled with Cell Trace Violet (Molecular Probes), and mixed with Tregs and irradiated (2000 rad) CD3^−^ PBMC, and then stimulated with anti-human CD3 OKT3 (eBioscience) in a V-bottom 96-well plate (20,000 cells/well of Teffs, indicated ratio of Tregs, 50,000/well of irradiated PBMC from which CD3^+^ cells were eliminated by human CD3^+^ T cell isolation kit (Stem Cells), and 0.1 μg/ml of anti-CD3 antibody). After 3 days of culture, cell proliferation was analyzed by flow cytometry. For the human transfected cells, anti-CD3/CD28 beads (0.02 μl/well, Thermo Fisher) were used instead of anti-CD3 and irradiated PBMC.

### iTreg Induction

Mouse CD4^+^ CD25^low^ CD45RB^high^ naïve T cells were sorted as described above. The cells were labeled with CTV, then seeded in a V-bottom 96-well plate (20,000 cells/well) and incubated with the indicated amount of TGFβ (Peprotec), 20 IU/ml of IL-2 and a 1:1 ratio of anti-CD3/CD28 magnetic beads (0.5 μl/well, Invitrogen) for 3 days. After the incubation, the cells were fixed and stained with anti-Foxp3-APC as described above, then analyzed using a FACS Canto (BD).

### IL-2 Promoter Assay

Jurkat cells (1,000,000 cells/ml) were transfected with IL-2 promoter (1.2 μg, Panomics), pRL-TK (0.03 μg, Promega), and indicated vectors (0.8 μg) using Fugene 6 per manufacturer's instructions. Twenty Four hours after transfection, cells were stimulated with PMA (50 ng/ml) and ionomycin (10 μM) for 6 h. A luciferase assay was then performed using Dual luciferase assay kit (Promega). Fold changes were calculated as (P.I. stimulated Firefly Luciferase/Renilla Luciferase)/(Non-stimulated Firefly lusciferase/Renilla Luciferase).

### Histology

Tissues were fixed with 10% neutral buffered formalin and embedded in paraffin. Sections were de-paraffinized and stained with H & E by the Cell Imaging Core of the Abramson Family Cancer Research Institute.

### Mouse *in vivo* Experiments

Six to Ten weeks old female Balb/c mice (*n* = 4 per group) were used for this set of experiments. Humanaized mAb 4D5 was obtained from Roche. For the *in vivo* treatment model, mice were randomly divided into groups and 5 × 10^5^ CT26Her2 cells were injected subcutaneously into both sides of the back of shaved mice. Treatments began a week later. 4D5 was injected IP (5 mg/kg) twice a week. DS-437 was injected (10 mg/kg) 5 times a week. Tumor size was measured with a digital caliper and calculated using a simple algorithm (3.14 × length × wide × height ÷ 6). For some experiments, 6–10 weeks old female MMTV-neu mice (*n* = 4 per group) were used. mAb 7.16.4 was purified from a hybridoma that was generated in our lab. For the *in vivo* treatment model, mice were randomly divided into groups and a rodent erbB2/neu transformed Balb/c breast tumor cell line, H2N113 (1 × 10^6^ cells) was injected subcutaneously into both sides of the back of mice. Treatments began 2 weeks later. 7.16.4 was injected IP (1.5 mg/kg) twice a week. EPZ004777 was injected (5 mg/kg or 25 mg/kg) 5 times a week. The experiments were not blinded but performed by a technician who wasn't knowledgeable about the expected outcome. The cell lines were tested for mycoplasma using a MycoSensor PCR Assay Kit (Agilent).

### Bisulfite Sequencing Analysis

Lymph node Tregs from Foxp3^Creyfp^ and Foxp3^Creyfp^-PRMT5^fl/fl^ mice (male, 18–21 days old) were collected using a FACSAria II as described above and then subjected to bisulfite sequencing analysis using the EZ-DNA Methylation Kit (Zymo Research) and primers described previously (18–20).

### Statistical Consideration

Statistical analysis was performed using the Student's *t*-test with unequal variance test using Microsoft Excel (one tail for *in vivo* TIL, two tail for others) for comparison of two groups. For 3 or more groups, one way ANOVA with Tukey HSD analysis were used for calculating the significance. At a minimum, data with a *p*-value < 0.05 were deemed significant. All *in vitro* experiments were performed in triplicate for calculating statistical significance. In all cases, experiments shown are representatives that were performed at least twice for *in vivo*, and 3 times for *in vitro* experiments.

## Results

### Identification of PRMT5 as a FOXP3 Binding Partner

**Figure 1:**
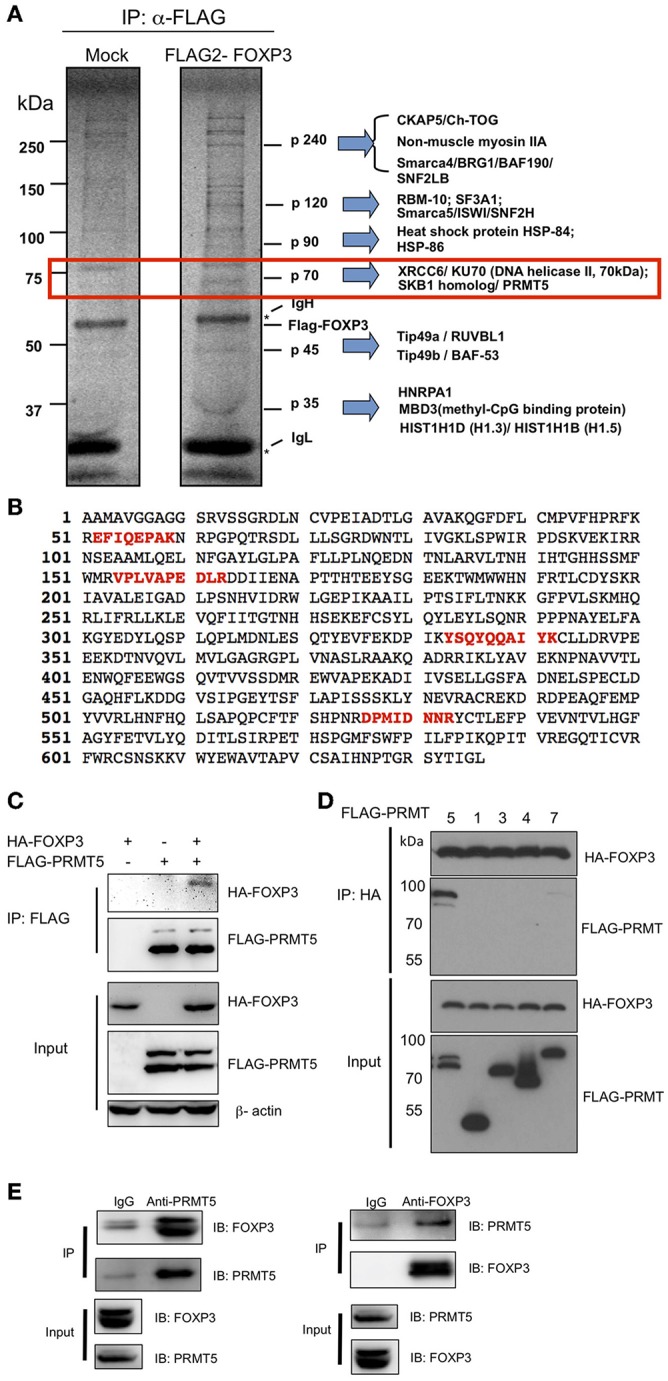
Identification of PRMT5 as a binding partner of FOXP3. **(A)** mass spectrum analysis of FOXP3 binding protein. 293T cells were transfected with pIPFLAG2-FOXP3 vector and immunoprecipitated with anti-FLAG agarose beads and subjected to SDS-PAGE. The proteins were visualized using a SilverQuest silver staining kit, and specific bands in FLAG-FOXP3 samples were analyzed by LC-MS/MS. **(B)** 4 peptide sequences of PRMT5 (labeled in red) were identified by MS sequencing as FOXP3 bound proteins. **(C,D)** specific interaction of PRMT5 with FOXP3 proteins. 293T cells were transfected with HA-FOXP3 and FLAG-PRMT5 **(C)** or HA-FOXP3 and FLAG-PRMT1, 3, 4, and 7 **(D)**. **(E)** endogenous interactions of FOXP3 with PRMT5 in human Tregs. Expanded human Tregs were lysed and subjected to immunoprecipitation with anti-PRMT5 (left panel) or anti-FOXP3 antibody (right panel). The samples were then blotted and protein detected with indicated antibodies.

To define the molecular interactions of FOXP3, we transfected and expressed FLAG-FOXP3 in 293T cells and then immunoprecipitated FOXP3 proteins with anti-FLAG agarose beads for identifying FOXP3-interacting proteins. We immunoprecipitated FOXP3 proteins with anti-FLAG agarose beads (Figure 1A). Several FOXP3 specific binding proteins were identified from the precipitation. Each specific band was extracted and subjected to mass spectrometry. One protein at ~70 kDa size was identified as the protein arginine methyltransferase (PRMT5) (Figure 1B). To verify the interaction between FOXP3 and PRMT5, we co-transfected HA-FOXP3 with FLAG-PRMT5 into 293T cells. As shown in Figure 1C, co-precipitation confirmed that FOXP3 binds to PRMT5. We further investigated whether other PRMT family members bind to FOXP3. We tested PRMT1, 2, 4, and 7 using 293T cells. PRMT1, 2, and 4 are know to be expressed in T cells (21), and PRMT7 is know to methylate arginine symmetrically (22). We found that PRMT5 preferentially binds to FOXP3 (Figure 1D). In addition, immunoprecipitates of anti-PRMT5 with human Treg lysate clearly included FOXP3 protein, and anti-FOXP3 precipitates included PRMT5 proteins (Figure 1E). These results indicate that PRMT5 is a binding partner of FOXP3 in Tregs.

### Deletion of PRMT5 in Tregs Causes Autoimmunity in Mice

**Figure 2:**
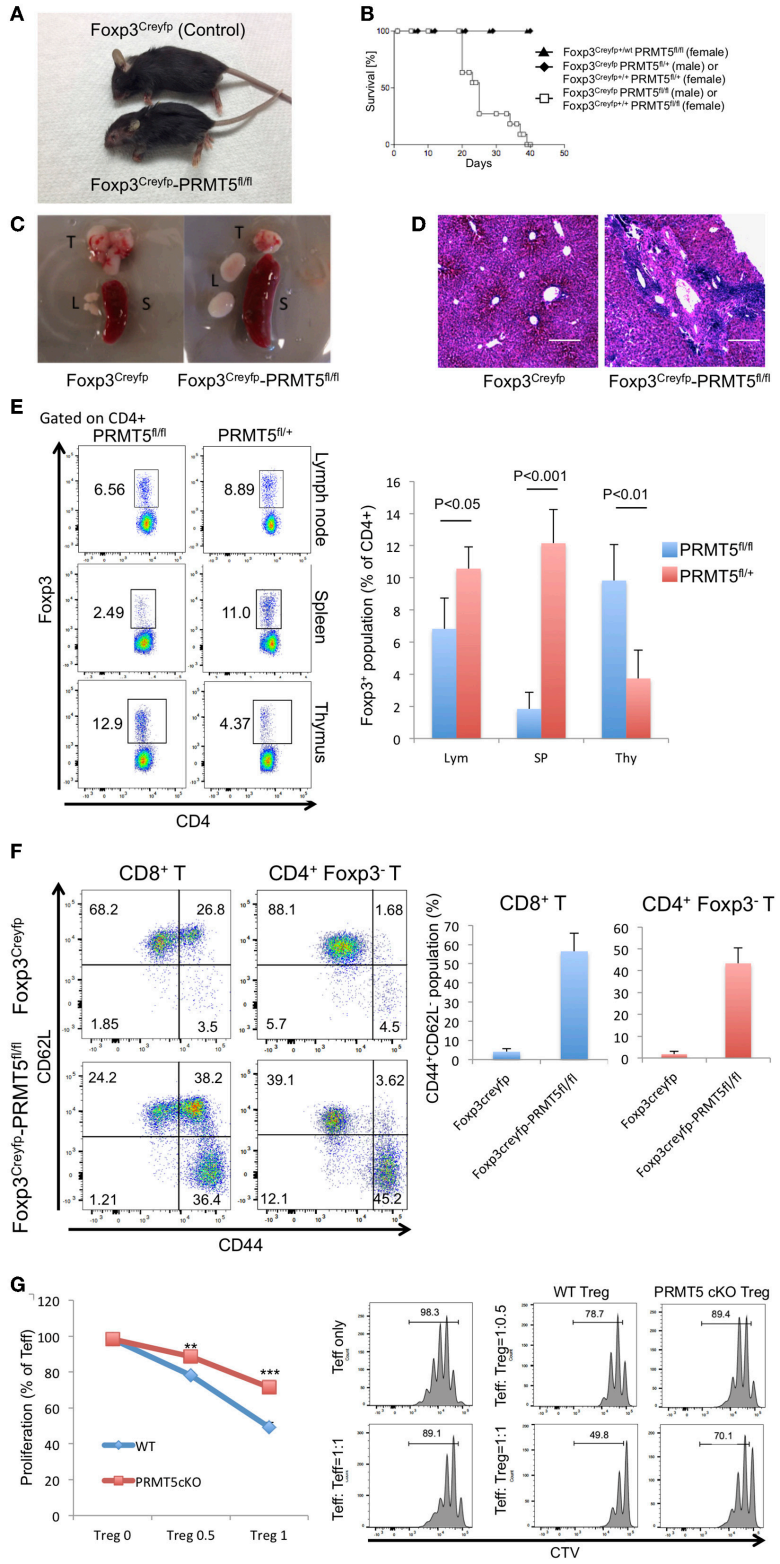
Foxp3^Creyfp^-PRMT5^fl/fl^ mice display scurfy-like symptoms. **(A)** phenotype of Foxp3^Creyfp^-PRMT5^fl/fl^ mouse (male, 18–21 days old). **(B)** survival curve of Foxp3^Creyfp^-PRMT5^fl/fl^ mouse (10 mice). **(C)** spleen (S), thymus (T), and lymph nodes (L) from Foxp3^Creyfp^ and Foxp3^Creyfp^-PRMT5^fl/fl^ mouse. **(D)** H&E staining of liver sections from Foxp3^Creyfp^ and Foxp3^Creyfp^-PRMT5^fl/fl^ mouse. Bar = 200 μm. Data shows a representative of 3 different mice. **(E)** Treg populations of spleen, lymph node and thymus from Foxp3^Creyfp^ and Foxp3^Creyfp^-PRMT5^fl/fl^ mouse Data shows a representative of 4 different mice. **(F)** activation of T cells in Foxp3^Creyfp^-PRMT5^fl/fl^ mice. TCRβ^+^ T cells from Foxp3^Creyfp^ and Foxp3^Creyfp^-PRMT5^fl/fl^ mouse lymph nodes (axillary and inguinal) were stained with anti-CD44 and CD62L antibodies, and analyzed by flow cytometry. Data shows a representative of 4 different mice. **(G)** suppressive functions of lymph node Tregs from Foxp3^Creyfp^ (WT) and Foxp3^Creyfp^-PRMT5^fl/fl^ (Prmt5 cKO) mouse. The error bars indicate the SD value. ^**^P < 0.005, ^***^P < 0.001 with Foxp3^Creyfp^ groups calculated by student *t*-test.

To reveal the role and function of PRMT5 on Tregs, we generated transgenic mice which have conditional PRMT5 deletion in Tregs since PRMT5 constitutive deficiency results in early embryonic lethality (23). We crossed PRMT5^fl/fl^ and Foxp3^Creyfp^ mice (11, 12) to produce PRMT5^fl/fl^ Foxp3^Creyfp^ mice. The transgenic mice that possess PRMT5 deficient Tregs displayed severe scurfy-like autoimmunity. Clinical features included weight loss, dermatitis and splenomegaly; and all animals died by 40 days after birth (Figures 2A–C). Necropsied livers revealed massive infiltration of lymphocytes as deduced by H&E sections (Figure 2D). This pathologic feature resembled those we observed in the liver of scurfy and Tip60^fl/fl^ Foxp3^Creyfp^ mice (12).

To study the Treg populations that still exist in the PRMT5^fl/fl^ Foxp3^Creyfp^ mice, we isolated CD4^+^ Foxp3^+^ YFP^+^ cells from spleen, lymph node and thymus (Figure 2E). Compared with control mice, PRMT5^fl/fl^ Foxp3^Creyfp^ mice possessed fewer Tregs in their spleens. However, the number of Tregs in peripheral lymph nodes was comparable. In the surviving Tregs, PRMT5 expression was dramatically deleted (Figure S1A), confirming conditional deletion in those Tregs. We observed increased Foxp3^+^ populations in the PRMT5^fl/fl^ Foxp3^Creyfp^ mouse thymus, which is reminiscent of Tip60^fl/fl^ Foxp3^Creyfp^ mice (12). This may be because massive inflammatory signaling induces Treg differentiation in the thymus.

We next investigated whether T cells in lymph nodes are activated and differentiated into the effector cells as deduced by staining for CD44 and CD62L. In the PRMT5^fl/fl^ Foxp3^Creyfp^ mice, the population of CD8^+^ and CD4^+^ Foxp3^−^ cells contained significant amounts of effector cells (the CD44^high^ CD62L^low^ population in Figure 2F), which is consistent with the severe scurfy phenotype. Also consistent with this observation, we found increased numbers of CD25^+^ cells in the CD4^+^ Foxp3^−^ and CD8^+^ population in the PRMT5^fl/fl^ Foxp3^Creyfp^ mice (Figure S1B), suggesting that PRMT5^fl/fl^ Foxp3^Creyfp^ mice have significantly increased amounts of activated T cells in their lymph nodes. In those T cell populations, we noted increased expression of IL-4 and IFNγ in the CD4^+^ Foxp3^−^ cells, and significant amounts of IFNγ^+^ cells in CD8^+^ T cells (Figure S1C), which is similar with the other scurfy-like phenotype reported previously (24).

We further analyzed the suppressive function of Tregs from lymph nodes by suppression assays. As shown in Figure 2G, PRMT5 deficient Tregs from PRMT5^fl/fl^ Foxp3^Creyfp^ mice had significantly less suppressive activity than Tregs from Foxp3^Creyfp^ littermates. We investigated if PRMT5 knockdown could also impair human Treg functions. Expanded human Tregs were transfected with lentivirus with or without shRNA targeting PRMT5. PRMT5 was successfully knocked down by shRNA in human Tregs (Figure S2A). Those Tregs showed impaired suppressive functions compared with empty vector transfected cells (Figure S2B), indicating PRMT5 is also important for human Treg function. These results indicate that a lack of PRMT5 in Tregs causes a reduction in Treg suppressive activity in cells that occupy the peripheral lymph nodes.

### Expression of Treg-Associated Molecules in PRMT5 cKO Tregs

We further investigated the molecules that are expressed in Tregs and associated with their function and maturation. In Tregs from PRMT5^fl/fl^ Foxp3^Creyfp^ mice, many function related surface markers such as CD25 and GITR were up-regulated (Figure S3A). Interestingly, Nrp-1 and Ki67 were dramatically decreased, compared with Foxp3^Creyfp^ mice. When we examined ICOS, the overall expression was increased, but the population of ICOS^high^ cells was decreased in PRMT5^fl/fl^ Foxp3^Creyfp^ mice. In addition, most of CD4^+^ Foxp3^+^ cells remained as CD44^low^ CD62L^high^ population compared with Foxp3^creyfp^ mice, suggesting that PRMT5 deletion prevents the maturation of Tregs as the effector/memory phenotype (Figure S3B).

**Figure 3:**
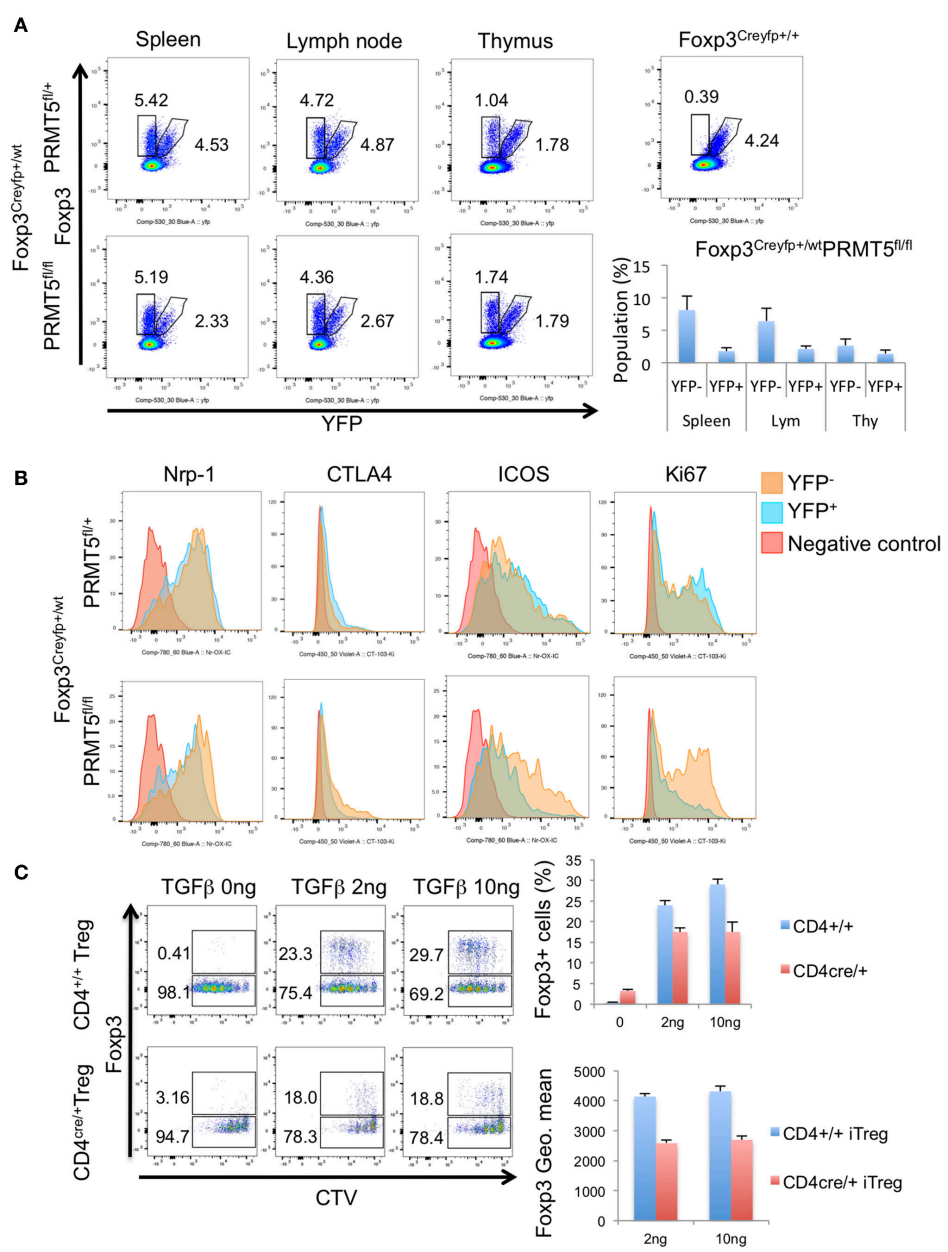
Populations and phenotypes of Tregs in Foxp3^Creyfp+/−^-PRMT5^fl/+^ or -PRMT5^fl/fl^ mice. **(A)** cells were harvested from spleen, lymph node, and thymus. Populations of YFP^+^ or YFP^−^ cells were analyzed. Numbers show the percentage of gated cells in CD4^+^ TCRβ^+^ cells. **(B)** phenotypes of Tregs. Each type of YFP^+^ or YFP^−^ cell was gated as **(A)**. Then expression levels of indicated proteins were analyzed. Data shows a representative of 3 different mice. **(C)** iTreg induction of CD4^+^T cells from CD4^cre^-PRMT5^fl/fl^ mice. CTV-labeled CD45RB^high^ CD25^low^ naïve CD4^+^T cells were treated with indicated concentrations of TGFβ, 20 IU/ml of IL-2 and 1:1 ratio of CD3/CD28 beads; then cultured for 3 days. After the incubation, cells were stained with anti-Foxp3 APC and analyzed by Flow cytometry.

To exclude the possibility that scurfy-like autoimmune phenotype affected the expression of these molecules, we used Foxp3^Creyfp^ heterozygous mice (Foxp3^Creyfphet^). In those heterozygous mice there is no scurfy-like phenotype as half of the Tregs are Foxp3^Creyfp^ positive because of random inactivation of the X chromosome (25). We observed a decreased number of YFP^+^ Tregs in PRMT5^fl/fl^ Foxp3^Creyfphet^ mouse spleen and lymph node (Figure 3A). In contrast, there were no notable differences between YFP^+^ and YFP^−^ Treg numbers in PRMT5^fl/+^ Foxp3^Creyfphet^ mice.

Next we compared the expression level of other known Treg-associated molecules. YFP^+^ cells from PRMT5^fl/fl^ Foxp3^Creyfphet^ mice showed decreased expression of Nrp-1, CTLA4, ICOS, and Ki67 (Figure 3B). These results indicate that PRMT5 deficient Tregs display both decreased suppressive and proliferative functions.

We also investigated whether PRMT5 deletion affects iTreg generation from naïve T cells by using CD4^cre^-PRMT5^fl/fl^ mice. PRMT5 deletion reduced the iTreg development compared with WT littermates (Figure 3C). In those iTregs, Foxp3 expression level was lower than WT. These results indicate that PRMT5 deletion also affects iTreg generation and Foxp3 expression.

### Gene Expression Profiles in PRMT5 Deleted Tregs

**Figure 4:**
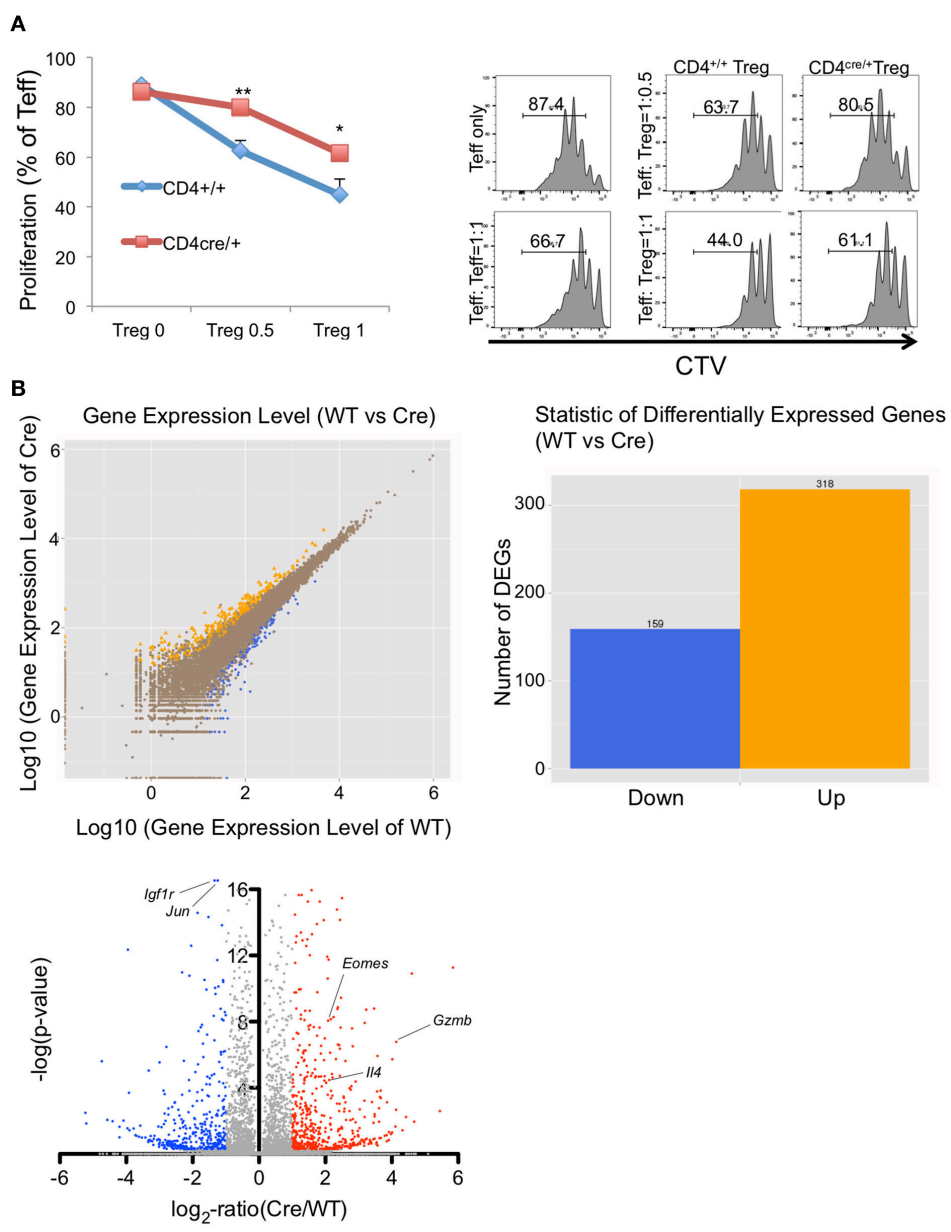
Functional and RNA sequencing analysis of PRMT5-deleted Tregs. **(A)** functional analysis of CD4^cre/+^ -PRMT5^fl/fl^ Tregs. CD4^+/+^ and CD4^cre/+^ -PRMT5^fl/fl^ Tregs were sorted as CD4^+^ CD25^high^ CD45RB^low^ cells and used for the Treg suppression assay as described above. CTV-labeled CD4^+^ CD25^low^ CD45^high^ cells from CD4^+/+^ PRMT5^fl/fl^ cells were used as effector cells. The error bars indicate the SD value. ^*^P < 0.05, ^**^P < 0.01 with CD4^+/+^ groups calculated by student *t*-test. **(B)** differential gene expression between WT (CD4^+/+^ -PRMT5^fl/fl^) and Cre (CD4^cre/+^-PRMT5^fl/fl^). Two mice per group were subjected for RNA sequencing analysis.

To reveal how PRMT5 deletion affects overall mRNA profiles expressed in Tregs, we collected PRMT5 deleted Tregs and subjected them to RNA sequencing analysis. For this experiment, we used the Tregs from CD4^+/+^ and CD4^cre/+^ -PRMT5^fl/fl^ mice because CD4^cre/+^ PRMT5^fl/fl^ mice do not display scurfy-like symptoms, principally because of reduced function of CD8^+^T and CD4^+^T cells (Tanaka et al. in preparation), to avoid the effect of endogenous inflammation in their bodies on their Tregs. In addition, CD4^cre^ enables an earlier deletion of PRMT5 expression in the T cells' developmental stage in the thymus that differs from Foxp3^cre^. This event possibly defines the complete phenotypic changes in generated Tregs. As shown in Figure 4A, Tregs from CD4^Cre/+^ showed significantly lower suppressive functions compare with WT Tregs. RNA sequencing analysis showed that 159 genes were down-regulated and 318 genes were up-regulated in the PRMT5 deleted Tregs (Figure 4B). Foxp3 induced genes, as reported by Kwon et al. (26), that include *igfr1* and *jun* were significantly decreased, and Foxp3 repressed genes such as *il18rap, Eomes, Gzmb*, and *il4* were up-regulated by PRMT5 deletion, (Table S1). Pathway analysis showed that PRMT5 deletion impacts a number of cell cycle pathway participating genes. Changes in p53, Foxo, and cytokine-cytokine receptor interaction pathways (Figure S4) were noted, supporting the notion that PRMT5 deletion causes both cell cycle abnormalities and induces an inflammatory phenotype in Tregs.

### Stability of PRMT5 cKO Tregs

Since we found a dramatic reduction of Tregs in Foxp3^creyfp^ PRMT5^fl/fl^ mouse spleen, we next studied the stability of PRMT5 deleted Tregs by analyzing epigenetic modifications of the CNS2 region, which mediates an important role in sustained expression of Foxp3 during Treg activation (27). To elucidate if a lack of PRMT5 can affect the methylation status in the Treg CNS2 region, we collected lymph node Tregs from Foxp3^Creyfp^ and PRMT5^fl/fl^ Foxp3^Creyfp^ mice and then analyzed their methylation status by bisulfite sequencing (Figure S5A). In the PRMT5-lacking Tregs, we noted that de-methylation of the CNS2 region was not observed, unlike wild type controls. We did not observe major alterations in the methylation status in the CNS1, CNS3, and upstream regions commonly hypomethylated and downstream hypermethylated regions. Consistent with this, proliferation of those Tregs resulted in the loss of Foxp3 expression (Figure S5B).

### Analysis of Symmetrical Dimethylation Sites on Foxp3

**Figure 5:**
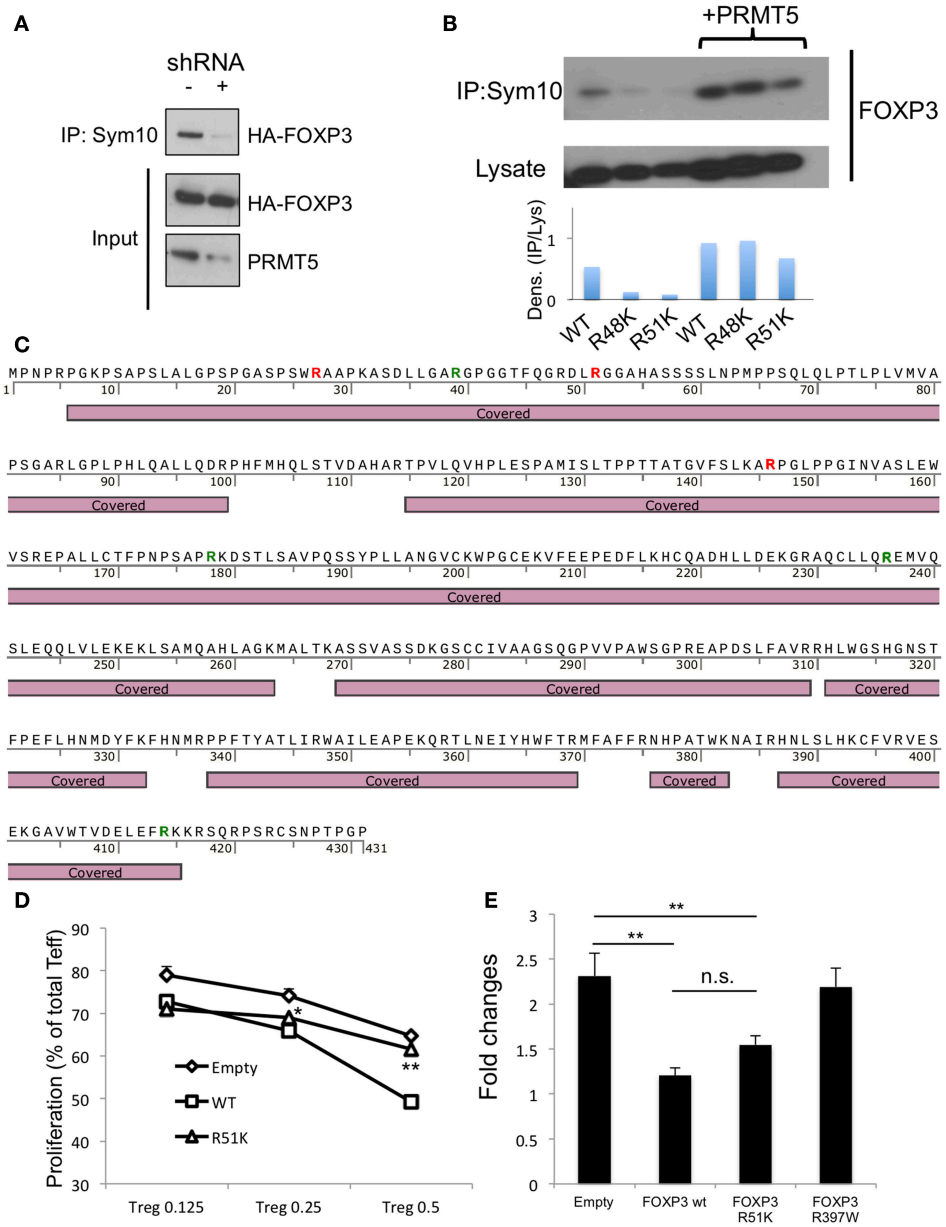
Analysis of methylation sites in human FOXP3. **(A)** symmetrical methylation of FOXP3. 293T cells were transfected with FOXP3 and empty or PRMT5 shRNA86 vector. After 24 h of transfection, the cells were lysed and immunoprecipitated with anti-sym10, which recognizes symmetrical arginine dimethylation, and then subjected to western blotting. **(B)** symmetrical methylation analysis of human FOXP3 wild type (WT), R48K and R51K mutant in 293T systems. **(C)** methylation analysis of FOXP3 by mass spectrometry. HA-FOXP3 transfected 293T cells were lysed and immunoprecipitated with anti-HA beads, then subjected for mass spectrometry. Covered: FOXP3 sequences detected by mass spectrometry. Red letters show the arginine that is di-methylated. Green letters show the arginine that is mono-methylated. **(D)** suppressive functions of FOXP3 R51K. Human CD4^+^ T cells were transfected with empty, FOXP3 WT and FOXP3 R51K mutant, then used as suppressors in the suppression assay. ^*^P < 0.05, ^**^P < 0.01 with FOXP3 WT groups calculated by student *t*-test. **(E)** IL-2 promoter assay with FOXP3-transfected Jurkat cells. Jurkat cells were transfected with the IL-2 promoter vector and additional indicated vectors. Twenty four hours after transfection, cells were transferred to 24 wells (250 μl/well), stimulated with PMA and ionomycin for 6 h, and then subjected to the luciferase assay. The error bars indicate the SD value. ^**^P < 0.01 with control vehicle groups calculated by one way ANOVA with Tukey HSD test.

To test whether FOXP3 can be methylated by PRMT5, we first analyzed if PRMT5 knockdown decreases symmetric arginine di-methylation signals by immunoprecipitation with anti-symmetric arginine di-methylation antibody sym10. PRMT5 deletion by shRNA (vector 86) could reduce PRMT5 expression, and symmetric arginine di-methylation signals (Figure 5A).

Next, we sought to identify the symmetric methylation sites. First we computationally analyzed the methylation sites on FOXP3 using the on- line web tool first reported by Kumar et al. (28). We found several possible methylation sites in Foxp3, including R51 and proximal R48 position. Consistent with this, Geoghegan et al. have suggested that FOXP3 can be di-methylated at the R51 position (21). However, Geoghegan and colleagues did not show whether that methylation is symmetric or asymmetric. We analyzed the effect of point mutation of FOXP3 R51 position to K on its symmetric methylation signals. R51K and proximal arginine R48K mutations both reduced endogenous FOXP3 methylation signals in 293T (Figure 5B). Over-expression of PRMT5 increased symmetric arginine di-methylation signals, but they were still lower in the FOXP3 R51K mutant, suggesting that position R51 is likely di-methylated by PRMT5.

We also confirmed by mass spectrometry that FOXP3 is di-methylated. Since 293T expresses high levels of PRMT5 proteins and is efficient for gene transfection, we used 293T transfected with HA-FOXP3 and purified the HA-FOXP3 using anti-HA magnetic beads. We found that there are several distinct dimethylation sites on FOXP3: R27 and R146, in addition to R51 (Figure 5C and Table S2).

We next analyzed the effect of the R51 point mutation on Foxp3 functions. Human CD4^+^ T cells were retrovirally transfected with the empty vector, FOXP3 WT or a mutated FOXP3 R51K gene and subjected to suppression assays. We found that the R51K mutation dramatically decrease its suppressive functions on T effector cells compare with FOXP3 WT (Figure 5D). We also analyzed if R51 mutation affects the direct binding of its target gene promoters by IL-2 promoter luciferase assay (Figure 5E).

The R51 mutation by itself did not alter FOXP3 suppressive activity on IL-2 promoter activity. On the other hand, the FOXP3 R397 mutation to W, which is known as a natural IPEX mutation and cannot bind to the promoter DNA sequences (29–31), did not display any suppressive function of IL-2 promoter. This result suggests that FOXP3 R51K mutation still has DNA binding ability.

### PRMT5 Is Important for Human Treg Functions

**Figure 6:**
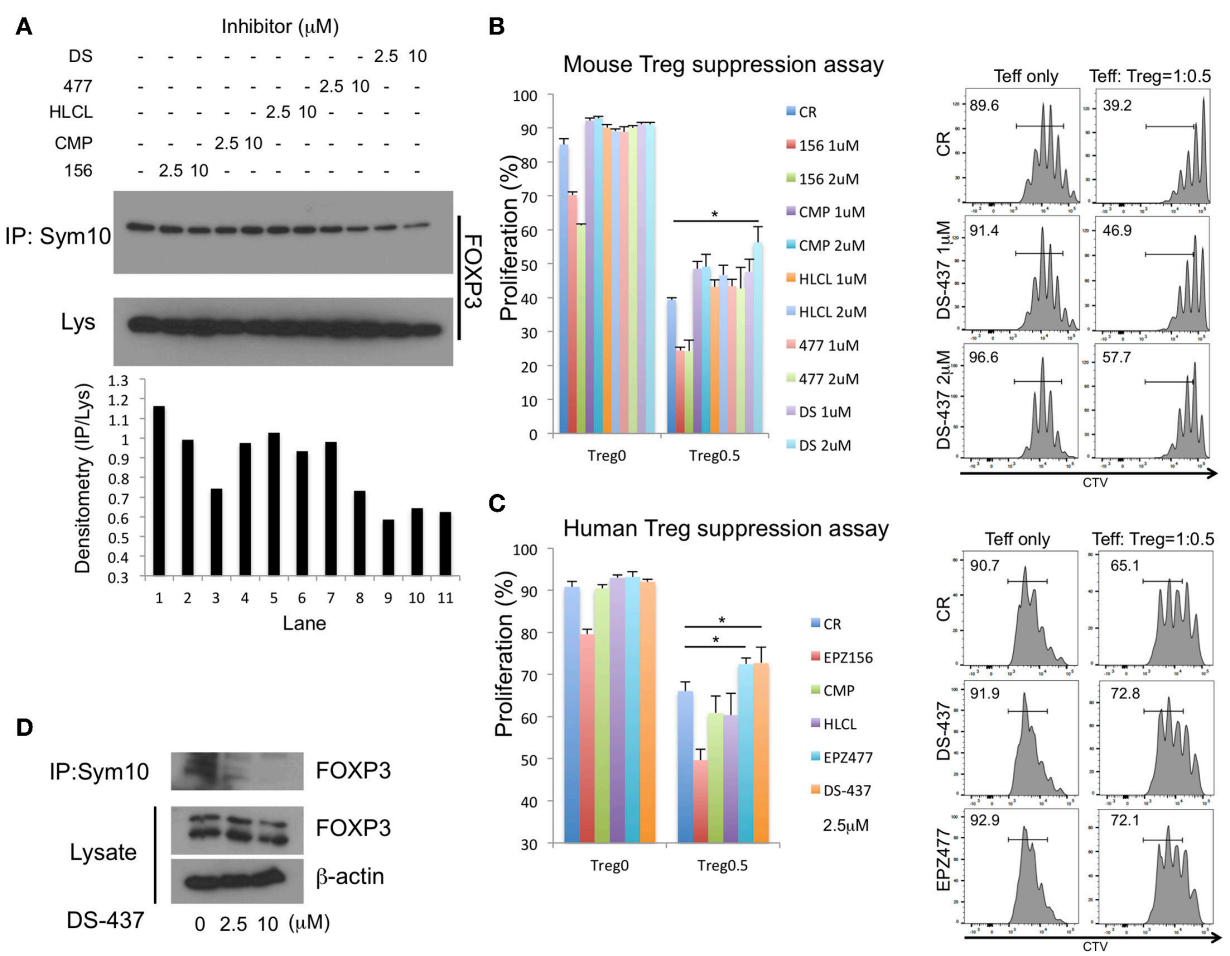
Effect of PRMT5 inhibitors on FOXP3 methylation and Treg function. **(A)** effect of PRMT5 inhibitors on FOXP3 methylation in 293T cells. 293T cells were transfected with FOXP3. After 24 h of transfection, the cells were incubated with indicated inhibitors for 16 h, then subjected to symmetrical dimethylation analysis as described above. 156: EPZ015666. CMP: CMP5. HLCL: HLCL61. 477: EPZ004777. DS: DS-437. **(B,C)** effect on suppressive function of mouse **(B)** and human **(C)** Treg suppression assay. The error bars indicate the SD value. ^*^P < 0.05 with control vehicle groups calculated by one way ANOVA with Tukey HSD test. **(D)** effect of PRMT5 inhibitor DS-437 on FOXP3 methylation in expanded human Tregs.

Pharmacologic PRMT5 inhibition should also impair Treg functions. There are several reported small molecule PRMT5 inhibitors including EPZ015666, CMP5, HLCL61, EPZ004777, and DS-437. DS-437 and EPZ004777 that are S-adenosylmethionine (SAM) competitive inhibitors (17, 32). CMP5 and HLCL-61 were computationally developed to bind the PRMT5 catalytic site, but they are not SAM-competitive inhibitors (33–35). EPZ015666 only binds to the complex of PRMT5, MEP50, and SAM (36), and MEP50 is a co-factor of PRMT5 (37). We initially investigated whether those inhibitors successfully inhibit FOXP3 methylation by 293T systems with anti-sym10 antibody. SAM-competitive inhibitor DS-437 and EPZ004777 showed better inhibition activities than other inhibitors, especially at 2.5 μM concentration (Figure 6A).

The effect of those inhibitors was examined in assays of Treg suppressive function to Teff cells. Treg suppression assays with mouse T cells revealed inhibition of mouse Treg functions with DS-437 (Figure 6B). EPZ015666 by itself led to strong inhibition of Teff proliferation (Figure 6B, red and green columns), while other inhibitors had no direct effect on Teff proliferation at the tested concentrations. EPZ004777, when used at 5 μM could inhibit mouse Treg functions (Figure S6A). This tendency was also noted in human Treg suppression assays (Figure 6C). In addition, DS-437 and EPZ00477 successfully inhibited endogenous FOXP3 methylation in the human expanded Tregs (Figure 6D and Figure S6B).

### SAM-competitive PRMT5 Inhibitors Enhance the Effect of Targeted Antibody Therapy in Murine Tumor Model

Our laboratory previously established many of the features of p185^erbB2/neu^ ectodomain targeted monoclonal antibody therapy. Our laboratory had also identified a contribution of Treg activity in dampening tumor elimination (5, 38), which has relevance to human breast tumor patients (7). To assess whether targeting PRMT5 could boost tumor immunity, we first employed the syngeneic MMTV-neu breast tumor model with EPZ004777 treatment, which is has previously been used in a mouse tumor model (32). Our preliminary experimental study found that EPZ004777 had better activity than EPZ015666 (Figure S6C). We next employed the combination of anti-erbB2/neu targeted therapy using the same model. Ten days after inoculation of syngeneic neu-transformed breast tumor cells (H2N113), mice were treated with anti-p185^erbB2/neu^ antibody 7.16.4, EPZ004777, or the combination of both. While we have noted that treatment with high dose EPZ004777 alone had modest beneficial effects on tumor growth inhibition (Figure S6D), even more effects were observed when mice were treated with the combination of EPZ004777 and the anti-p185^erbB2/neu^ antibody. Interestingly, the 7.16.4 group alone had the highest Treg levels among TILs (Figure S6E). Importantly, EPZ004777 treatment lessened 7.16.4 induced Treg infiltration into tumors.

**Figure 7:**
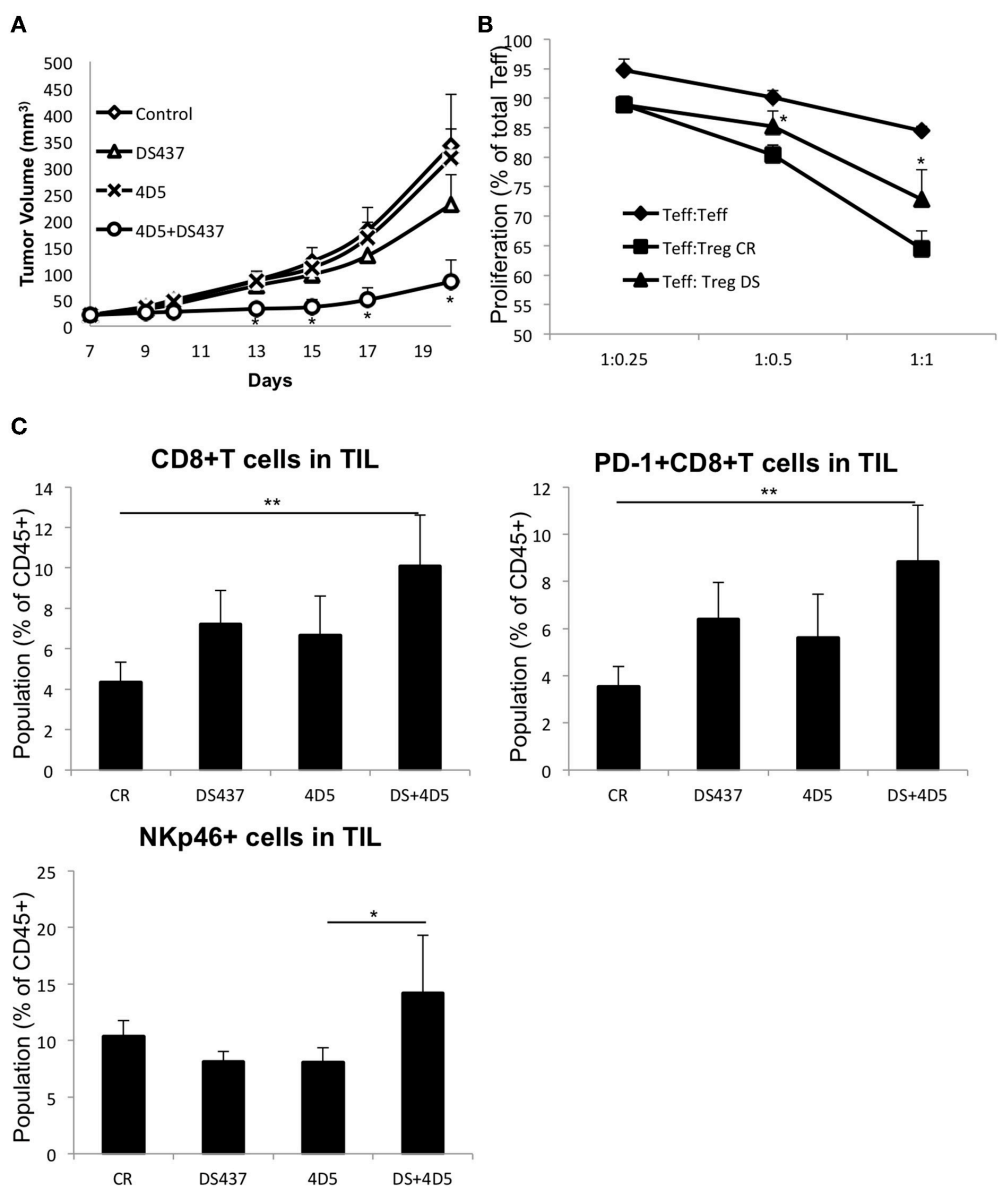
Effect of PRMT5 inhibitor DS-437 on targeted therapy. **(A)** effect of DS-437 combined with anti-erbB2 antibody 4D5 on tumor size in CT26Her2 tumor model. CT26Her2 tumor cells (0.5 × 10^6^) were injected subcutaneously into Balb/c mice that were treated with control PBS, 4D5 (5 mg/kg, twice per week), DS-437 (10 mg/kg, 5 times per week), or both 4D5 and DS-437. Data represent mean + SEM. One way ANOVA with Tukey HSD test was performed to compare the difference in the tumor size of different treatment groups. **(B)** function of Tregs in tumor bearing mice. Teffs and Tregs of PBS (CR) or DS-437 (DS) treated mouse lymph nodes were collected by sorting as described above. Teffs were labeled with CTV and stimulated with dynabeads CD3/CD28 beads (0.2 μl/well) with or without Tregs as indicated ratios. After 3 days of culture, proliferation of Teffs were analyzed by flow cytometry. The error bars indicate the SD value. ^*^P < 0.05 with control groups calculated by student *t*-test. **(C)** TIL were obtained after treatment as in **(A)** and the cell population analyzed by flow cytometry (4 mice per group, one tumor from each mouse). One way ANOVA with Tukey HSD test was performed to compare the difference in the populations of TIL of different groups. ^**^P < 0.01 (CR vs. DS+4D5); ^*^P < 0.05 (4D5 vs. DS+4D5).

We next sought to determine whether inhibition of PRMT5 could improve anti-p185^erbB2/neu^ targeted therapy in the resistant tumor model. CT26-Her2 cells were used. CT26 cells were established by engineering the Balb/c syngeneic tumor line CT26 to express human Her2 (39) as a naturally resistant model for anti-p185^erbB2^ antibody therapies as it carries the oncogenic K-RasG12D mutation (40, 41). Although the animals treated with DS-437 alone had some beneficial effects on inhibiting tumor growth (Figure 7A), the combination of DS-437 and the anti-p185^erbB2/neu^ antibody 4D5 had even more dramatic effects (Figure 7A). Remarkably, only 3/8 tumors continued to grow in the combination group mice (Figure S7A). 4D5 treatment by itself did not show a beneficial effect in this model. The mice treated with DS-437 showed significantly lower Treg activities compare with PBS-treated mice (Figure 7B). We further analyzed tumor infiltrating lymphocytes (TILs) in the treated mice (Figure S7B). In the DS-437 treated groups, there are increased total CD8^+^ and CD8^+^ PD-1^+^ T cells, but more significantly in combination groups compared with controls (Figure 7C). Interestingly, the combination groups had significantly increased NKp46^+^ cells in their tumors. We also noted that there are not significant changes of the expression of CD206 in the F4/80^+^ macrophage population from combination groups (Figure S7C).

## Discussion

In this study, we defined a FOXP3 post-translational modifier, PRMT5. As far as we know, this is the first report that Foxp3 interacts with and can be methylated by PRMT5. Acuto et al. have shown that FOXP3 is methylated at R51, however they did not specify which member of the PRMT family methylates FOXP3 (21). Mass spectrometry analysis revealed that there are several di-methylated arginines in FOXP3. We found that the FOXP3 R51 position is di-methylated and appears to be symmetrically di-methylated by PRMT5. The point mutation of FOXP3 at R51 did not alter IL-2 promoter binding, but decreased the suppressive function of transduced CD4^+^T cells, suggesting FOXP3 methylation does not alter DNA binding by itself, but has a role in FOXP3 suppressive functions, and di-methylation at R51 is important for Treg function.

Post-translational arginine modification of proteins is known to regulate many biological processes. PRMT proteins transfer methyl groups from SAM to arginine residues. There are 3 types of PRMTs; and PRMT5 belongs to the Type 2 PRMT set, which can symmetrically di-methylate arginine residues of target proteins (37). Although there are some reports identifying PRMT7 as a Type 2 methylation protein (22), our data showed that PRMT7 does not interact with FOXP3, supporting our supposition that PRMT5 is the dominant protein that can methylate FOXP3 symmetrically.

PRMT5 proteins were identified as histone methylating enzymes that led to suppression of gene expression. However, more recently, PRMT5 has been found to methylate and alter the activity of transcription factors. Di-methylation of the NF-kB p65 subunit enhances its function (42), while methylation of the tumor suppressor genes p53 and E2F1 attenuates their activity (22). Our results indicate that PRMT5 is indispensable for normal Treg activity, which prevents the severe autoimmunity scurfy-like symptoms. Interestingly, although those PRMT5 cKO Tregs show defective functions and less expression of CTLA4, Nrp-1, and ICOS, expressions of other important molecules for Treg suppressive functions such as GITR, OX40, and Helios are still comparable to normal (data not shown). We also found that PRMT5 deleted Tregs show less activation as demonstrated by dramatic loss of the CD44^high^ CD62L^low^ population. In addition to CD44 and CD62L, ICOS expression in Tregs is also known to reflect effector and memory phenotype of Tregs and has strong immune suppressive functions (43–46). These results suggest that PRMT5 deletion prevents Treg cell progression to effector/memory phenotypes, and that may have a role in the strong scurfy-like symptoms in Foxp3^cre^-PRMT5^fl/fl^ mice.

RNA sequencing results and Ki-67 staining clearly indicate that PRMT5 deletion affects Treg proliferation. In addition, those Tregs show increased inflammatory cytokine and cytokine receptor expression. These results also suggest that PRMT5 deletion weakens the Foxp3 suppressive function of expression of those inflammatory cytokines. Indeed, several Foxp3 regulating genes reported by Kwon et al. (26) are dramatically changed in the PRMT5 deleted Tregs. Interestingly, *igfr1*, which is up-regulated by Foxp3 (26) and is important for Treg proliferation and function (47–49), is significantly decreased in the PRMT5 deleted Tregs. Reduction of *igfr1* may also influence the decrease of Treg proliferation in PRMT5^fl/fl^ Foxp3^Creyfp^ mice.

Zhao et al. showed that the symmetrical methylation of histone H4 at R3 position specifically recruits the DNA methyltransferase DNMT3a to suppress the targeted genes (50, 51). Other studies were unable to verify that interaction (52). To identify the effect of PRMT5 deletion on Treg CNS methylation status, we also examined direct DNA methylation. Our study showed that the PRMT5 deletion was associated with hypermethylation in the CNS2 region, suggesting that the Histone H4R3-mediated DNMT3a recruitment by PRMT5 does not affect the methylation status of CNS2 region. While CNS2 hypermethylation may contribute to a less proliferative phenotype, PRMT5 cKO Tregs suffer from less stable Foxp3 expression and expansion in the periphery. Fang et al. have shown that de-methylation of CNS2 sustains Foxp3 expression in mature proliferating Tregs (27). Nevertheless, mice whose CNS2 region has been deleted in a Foxp3-dependent manner do not display severe scurfy-like symptoms as seen with the PRMT5 cKO mice we describe herein.

In our experimental conditions, we have not noted increased expression of Foxp3 by inhibiting PRMT5. Those studies include genetic knock outs, shRNA, and inhibitor experiments in both mice and humans. On the other hand, a recent publication by Zheng et al. found that PRMT5 negatively regulates FOXP3 expression in human Tregs, especially in ulcerative colitis patients (53). However, Zheng et al. used the AMI-1 inhibitor, which is known to inhibit PRMT1, 2, 4, and 6. AMI-1 does not selectively effect PRMT5 (54). In addition, the Zheng studies did not clearly demonstrate shRNA effects. It is possible that PRMT5 inhibition decreases inflammatory disease. The substrate (e.g., histone H3) competitive inhibitor EPZ015666, which acts as an inhibitor of H3 methylation but is not effective for inhibiting Foxp3 methylation, strongly inhibits Teff cell proliferation. The decrease of PRMT5 seen in the shRNA experiment in the Zheng et al. studies, might demonstrate effects on histone methylation.

PRMT5 is known to be expressed universally but is overexpressed in a large number of cancers, including breast cancer (37, 55). Thus, there are efforts to develop pharmaceutical inhibitors for this protein. CMP5, which was developed by computational simulation, showed suppressive function of Th1 cells at high doses (56). In our experiments, EPZ015666 showed significant inhibition of Teff cell proliferation, which we consider as disadvantageous for tumor therapy. We found that SAM-competitive inhibitors efficiently inhibit Foxp3 methylation and do not inhibit Teff cell proliferation, at least at the concentration we tested. These results indicate that a SAM-competitive PRMT5 inhibitor may be a better candidate because of its activities in both malignant phenotype inhibition and induction of tumor immunity. Consistent with this, we found better activity of EPZ004777 than EPZ015666 on our syngeneic tumor models (data not shown).

Our results suggest that treatment with a PRMT5 inhibitor in a syngeneic mouse erbB2/neu breast tumor model shows only a slight effect on tumor growth by itself, but significantly enhanced anti-erbB2 targeted antibody therapy. This model only demonstrated a small number of tumor-infiltrating Tregs; but, importantly and unexpectedly, the level of Tregs actually increases with targeted antibody treatment. This inhibitor limits antibody-dependent Treg infiltration.

In human patients, increased frequency of Tregs during trastuzumab therapy coincided with disease progression (57, 58). In addition, recent clinical observations indicate that early-stage HER2-positive breast cancers that did not react with neoadjuvant combinations such as docetaxel, carboplatin, and trastuzumab with or without pertuzumab demonstrate an immunosuppressive tumor microenvironment phenotypes, and this phenotypic change involves CD4^+^ and FoxP3^+^ cells that represent the Treg population (59). Thus, targeting Tregs during anti-p185^erbB2^ targeted therapy may represent a therapeutic benefit to erbB2 driven breast cancer patients.

We did not see increased Tregs in humanized 4D5-treated tumors in our CT26Her2 tumor model (data not shown). This difference probably arises because CT26Her2 malignant growth is driven by the RAS oncogene and resistant to erbB2/neu targeted therapy. Rather, we observed inhibited Treg functions in DS-437 treated mouse lymph nodes and increased total CD8^+^ T cells, CD8^+^ PD-1^+^ T cells, and NKp46^+^ cells in their TIL. CD8^+^ PD-1^+^ T cells are known to be tumor-reactive T cells (60), indicating that DS-437 treatment induced tumor-specific immunity in those mice.

We found that the combination of 4D5 and DS-437 also increased the NKp46^+^ cell population. NKp46 is a marker of NK cells, suggesting that the combination therapy also induced NK cell activity. We noted DS-437 does not affect the tumor associated macrophage's phenotype, suggesting that PRMT5 inhibition during anti-erbB2 mAb therapy would not be dependent on the macrophage activity.

Our studies identify PRMT5 as a FOXP3 binding partner and epigenetic modulator, and potentially important for Treg-targeting small molecules. PRMT5 deletion in Tregs caused scurfy-like symptoms and severe autoimmunity in the mice. PRMT5 deleted Tregs clearly showed proliferation abnormality and decreased suppressive functions. In this study we used many different inhibitors of PRMT5. Most of the inhibitors appeared to inhibit FOXP3 methylation. However, DS-437 and EPZ004777, which are SAM competitive inhibitors, displayed the most efficient suppression of FOXP3 methylation and greater activity in limiting Treg function.

Our experiments indicate that limiting PRMT5 function may promote tumor immunity by inhibiting Treg function and limiting Treg migration into tumors. Interestingly, PRMT5 levels and activity is reported to be elevated in several tumor cells and abnormal PRMT5 functions may contribute to some aspects of the malignant phenotype (37). Therefore, targeting PRMT5 coupled with targeted therapy represents a rational strategy for both cancer immunotherapy and tumor-targeted therapy.

## Author Contributions

YN performed *in vivo* and *in vitro* experiments, interpreted the results and wrote manuscript. MJ performed *in vivo* tumor study. FZ performed endogenous interaction of Foxp3 with PRMT5 in human Tregs. YX made Foxp3 constructs. YT and TK generated CD4^cre^-PRMT5^fl/fl^ mice. SF helped in the conceptual organization of the paper. MMG initiated efforts to define the role of tumor localized myeloid cells in the erbB2/neu targeted therapy. BL performed mass spectrometry and discovered Foxp3-PRMT5 interaction. TO generated Foxp3^creyfp^-PRMT5fl/fl mice and performed bisulfite sequencing. HZ helped to design the *in vivo* tumor experiments. MIG wrote manuscript, interpreted the results, designed experiments and organized this project.

### Conflict of interest Statement

MMG, is an employee and shareholder in Macrophage Therapeutics, MIG, is an advisor to Macrophage Therapeutics. The remaining authors declare that the research was conducted in the absence of any commercial or financial relationships that could be construed as a potential conflict of interest.
