# Supplementary material for: PRMT5 Associates With the FOXP3 Homomer and When Disabled Enhances Targeted p185erbB2/neu Tumor Immunotherapy
Source: Front Immunol. 2019 Feb 8;10:174. doi: 10.3389/fimmu.2019.00174 (PMC6375878; doi:10.3389/fimmu.2019.00174)
Supplement: Supplementary file 3 [file Data_Sheet_1.docx]

Fig. S1. Phenotype of mouse Foxp3^Creyfp^-PRMT5^fl/fl^ T cells. (**A**), lymph node Teff (TCRβ^+^ CD4^+^ CD25^low^ CD45RB^high^ cells) and Tregs (TCRβ^+^ CD4^+^ CD25^high^ CD45RB^low^ YFP^+^ cells) were sorted from Foxp3^Creyfp^ (WT) and Foxp3^Creyfp^-PRMT5^fl/fl^ (PRMT5 cKO) mice. Cells were lysed and subjected to western blotting with indicated antibodies. (**B**), population of CD25^+^ cells in CD4^+^ Foxp3^-^ and CD8^+^ T cells in Foxp3^Creyfp^ and Foxp3^Creyfp^-PRMT5^fl/fl^ mouse spleens. (**C**), Cytokine expression of CD4^+^ and CD8^+^ T cells from Foxp3^Creyfp^ and Foxp3^Creyfp^-PRMT5^fl/fl^ mouse spleens.

** Fig. S2.** Deletion of PRMT5 in human Tregs. (**A**), effect of shRNA targeting PRMT5 in human Tregs. Expanded human Tregs were transfected with lentiviral shRNA vectors and then selected with puromycin. After 6 days of culture, cells were harvested and lysates subjected to western blotting with indicated antibodies. (**B**), function of PRMT5 knock-down human Tregs. Cells transfected with empty or shRNA targeting PRMT5 (shPRMT5 86) were collected and subjected to an *in vitro* suppression assay. The error bars indicate the SD value. Significance was calculated by student t-test.

**Fig. S3.** Phenotypes of PRMT5cKO Tregs. (**A**), Tregs were gated as (TCRβ^+^ CD4^+^ CD25^high^ CD45RB^low^ YFP^+^ cells), and then expression levels of indicated proteins were analyzed by flow cytometry. (**B**), memory/effctor phenotype of Tregs. Cells were stained with anti-CD44 and CD62L antibody and expression levels were analyzed by flow cytometry. Data shown are representative of 4 different mice.

**Fig. S4.** Scatter plot of KEGG pathway enrichiment statistics of RNA sequencing.

**Fig. S5.** PRMT5cKO Tregs show unstable phenotype. (**A**), bisulfite Sequencing analysis of lymph node Tregs from Foxp3^Creyfp^ and Foxp3^Creyfp^-PRMT5^fl/fl^ mice. (**B**), expanded Tregs from Foxp3^Creyfp^-PRMT5^fl/fl^ mice lose Foxp3 expression. Tregs from Foxp3^Cre^ and Foxp3^Creyfp^-PRMT5^fl/fl^ mouse lymph nodes were cultured with IL-2 (200 IU/ml) and CD3/CD28 dynabeads (1:1) for indicated days and then analyzed for Foxp3 expression by flow cytometry.

**Fig. S6.** Effect of SAM-competitive PRMT5 inhibitor EPZ004777. (**A**), Treg suppression assay of mouse T cells with PRMT5 inhibitor EPZ004777 with higher doses. The error bars indicate the SD value. (**B**), effect of PRMT5 inhibitor EPZ004777 on endogenous Foxp3 methylation in expanded human Tregs. (C), effect of PRMT5 inhibitor EPZ015666 and EPZ00477 on syngeneic mouse MMTV-neu breast tumor model. H2N113 tumor cells (1 x 10^6^) were injected subcutaneously into MMTV-neu mice that were treated with control PBS, EPZ015666 or EPZ004777 (25mg/kg, 3 times/week). Data represent mean + SEM. One way ANOVA with Tukey HSD test was performed to compare the difference. (**D**), effect of EPZ004777 combined with anti-erbB2 antibody on tumor size in syngeneic mouse MMTV-neu breast tumor model. H2N113 tumor cells (1 x 10^6^) were injected subcutaneously into MMTV-neu mice that were treated with control PBS, 7.16.4 (1.5 mg/kg, twice per week) and EPZ004777 low (04777 (L), 5 mg/kg, 5 times per week), EPZ004777 high (04777 (H), 25 mg/kg, 5 times per week), and 7.16.4 and EPZ004777 high. Data represent mean + SEM. One way ANOVA with Tukey HSD test was performed to compare the difference. ^*^*P*< 0.05, ^**^*P*< 0.01 compared with control. (**E**), population of Foxp3^+^ CD4^+^ T cells in TIL. One way ANOVA with Tukey HSD test was performed to compare the difference. ^*^*P*< 0.05.

**Fig. S7.** Tumor analysis described in Fig. 7. (**A**), individual tumor growth. (**B**), gating strategy of TIL analysis by flow cytometery. (C), CD206 expression of tumor infiltrated macrophages. TIL were obtained after treatment as in Fig.7A and the F4/80^+^ population were analyzed by flow cytometry.
